# DNA binding fluorescent proteins for the direct visualization of large DNA molecules

**DOI:** 10.1093/nar/gkv834

**Published:** 2015-08-11

**Authors:** Seonghyun Lee, Yeeun Oh, Jungyoon Lee, Sojeong Choe, Sangyong Lim, Hyun Soo Lee, Kyubong Jo, David C. Schwartz

**Affiliations:** 1 Department of Chemistry and Interdisciplinary Program of Integrated Biotechnology, Sogang University, 1 Shinsudong, Mapogu, Seoul, 121-742, Korea; 2 Research Division for Biotechnology, Korea Atomic Energy Research Institute, Jeongeup 580-185, Korea; 3 Laboratory for Molecular and Computational Genomics, Department of Chemistry, Laboratory of Genetics, University of Wisconsin-Madison, Madison, WI 53705, USA

## Abstract

Fluorescent proteins that also bind DNA molecules are useful reagents for a broad range of biological applications because they can be optically localized and tracked within cells, or provide versatile labels for *in vitro* experiments. We report a novel design for a fluorescent, DNA-binding protein (FP-DBP) that completely ‘paints’ entire DNA molecules, whereby sequence-independent DNA binding is accomplished by linking a fluorescent protein to two small peptides (KWKWKKA) using lysine for binding to the DNA phosphates, and tryptophan for intercalating between DNA bases. Importantly, this ubiquitous binding motif enables fluorescent proteins (*K*_d_ = 14.7 μM) to confluently stain DNA molecules and such binding is reversible via pH shifts. These proteins offer useful robust advantages for single DNA molecule studies: lack of fluorophore mediated photocleavage and staining that does not perturb polymer contour lengths. Accordingly, we demonstrate confluent staining of naked DNA molecules presented within microfluidic devices, or localized within live bacterial cells.

## INTRODUCTION

Genetically engineered fluorescent proteins (FPs) have revolutionized the fields of cell and molecular biology, because FP-tagging allows specific proteins to be directly visualized, in real time, for the elucidation of binding patterns and dynamics within living cells. FP-tagged proteins are usually expressed within cells and this feature leverages genetic regulation not accessible using synthetic dyes, or quantum dots (1). *In vitro* applications include imaging of single proteins bound to DNA molecules for studying localization and dynamic behaviour (2).

DNA molecules and chromosomes are typically stained using fluorescent organic dyes; for example, ethidium bromide (EtBr) is commonly used for staining DNA after gel electrophoresis. Large DNA molecules are often imaged by fluorescence microscopy using a bis-intercalating dye of an oxazole yellow homodimer (YOYO-1) (3) and DNA within viable cells is stained with membrane permeable dyes, such as SYTO (4). In addition, a number of new approaches have recently introduced alternative ways of visualizing DNA molecules with organic fluorescent dyes (5,6) using nick translation (7,8), click chemistry (9), photochemical reaction with photolabile protecting groups (10), and sequence specific labelling (11). However, these organic dyes present numerous fundamental drawbacks for staining DNA molecules, or chromosomes (12): (i) Many DNA-staining organic dyes are cytotoxic; consequently, they are potential mutagens, requiring careful handling (13,14). (ii) Organic dyes synergize DNA photodamage under laser illumination through formation of radical intermediates that create single, or double stranded breaks in DNA molecules and trigger ‘phototoxicity’ in living cells (15). (iii) Organic dyes bleach and dim under continuous illumination during imaging and are not readily replenished for restoring full luminosity at binding sites by fresh, unbleached fluors.

Given these concerns, FPs are promising candidates for staining DNA, especially when considering that the quantum yield of eGFP (0.66) is higher than YOYO-1 (0.52) and EtBr (0.15) (16,17). Nevertheless, DNA molecules have never been confluently stained using FPs, both in *in vivo* and *in vitro* experiments for lack of a suitable DNA binding motif. Accordingly, our goal here was to create a FP tag with a short DNA-binding peptide for evenly staining DNA molecules in ways that would support both *in vitro* and *in vivo* applications, yet maintain the inherent advantages of FPs over fluorescent organic dyes. Here, we report the development of a novel DNA-staining FP (FP-DBP) that allows visualization of elongated large DNA molecules within microfluidic devices and nucleoid localization within live bacterial cells.

## MATERIALS AND METHODS

### Chemicals

All DNA primers and oligonucleotides were purchased from Bioneer (Daejeon, Korea). All enzymes were purchased from New England Biolabs (Ipswich, MA, USA). λ DNA (48.5 kb) and 1 kb DNA ladder were purchased from Bioneer (Daejeon, Korea) and T4 GT7 DNA (165 644 bp) was purchased from Nippon Gene (Tokyo, Japan). *Escherichia coli* strain BL21 (DE3) was purchased from Yeastern (Taipei, Taiwan). Biotin-labeled bovine serum albumin (BSA) was from Sigma (St. Louise, MI, USA), neutravidin was from Pierce (Rockford, IL, USA), and BSA was from New England Biolabs (Ipswich, MA, USA). Epoxy was from Devcon (Riviera Beach, FL, USA). *N*-trimethoxymethyl silyl propyl-*N*,*N*,*N*-trimethyl ammonium chloride in 50% methanol was purchased from Gelest (Morrisville, PA, USA). Ni-NTA agarose resin and disposable column (empty gravity column) were purchased from Qiagen (Venlo, Netherlands). Non-SDS TBE-PAG was purchased from Komabiotech (Seoul, Korea). Other chemicals were from Sigma.

### Microscopy

The microscopy system consisted of an inverted microscope (Olympus IX70, Tokyo, Japan) equipped with 60× and 100× Olympus UPlanSApo oil immersion objectives, illuminated using a solid-state laser (LBX488 and SLIM 532, Oxxius, Lannion, France). The laser light was focused on the multimode optical fiber (BFH-22-550, Thorlabs, Newton, NJ, USA) and passed through a 488 and 532 nm holographic notch filter (Semrock, Rochester, NY, USA), which was installed to prevent laser lights from reaching the EMCCD camera. For DAPI staining, 60× Olympus UCPLFLN objectives, 100 W mercury lamp (U-LH100HG, Olympus, Tokyo, Japan) and corresponding filter sets were used. Fluorescence images were captured by an electron-multiplying charge-coupled device digital camera (Qimaging Rolera EM-C^2^, Surrey, BC, Canada) and stored as 16 bit TIFF format generated by the software Image Pro Plus (Media Cybernetics, Rockville, MD, USA). For image processing and length measurements, ImageJ was utilized with Java plug-ins developed in our lab.

### Protein construction, purification and cell growth

**Figure 1.**
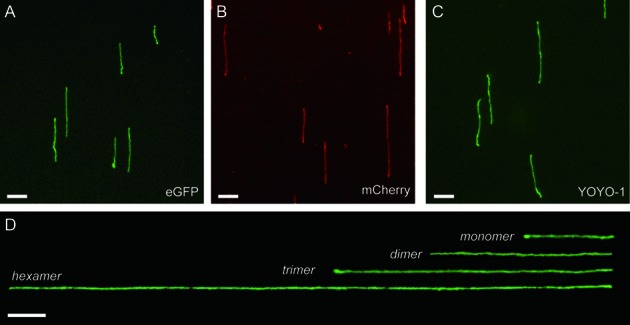
Large, stretched DNA molecules stained with FP-DBPs. λ DNA (48.5 kb) stained with (**A**) FP(eGFP)-DBP, (**B**) FP(mCherry)-DBP, (**C**) YOYO-1 for comparison, (**D**) λ DNA concatemers stained by FP(eGFP)-DBP. The full sequence of FP(eGFP)-DBP is MGSSHHHHHHSSGLVPAGSH-KWKWKKA-eGFP-AKKWKWK. Scale bars = 5 μm.

The plasmid pET-15b (Novagen, Germany) was used for the transformation and expression of FP(eGFP)-DBP. For tagging DNA binding parts to each terminals of eGFP, forward primer (5′-ATG TTG CAT ATG ***AAA TGG AAA TGG AAA AAA GCG*** ATG CGT GAG CAA GGG CGA GGA GC-3′, N’-***KWKWKKA***-C’) and reverse primer (5′-ATG TTG GGA TCC TTA ***TTT CCA TTT CCA TTT TTT*** *CGC* CTT GTA CAG CTC GTC CAT GCC-3′, N’-***AKKWKWK***-C’) were used in the PCR process. For FP(mCherry)-DBP, forward primer (5′-ATA TTG CAT ATG AAA TGG AAA TGG AAA AAA GCG ATG GTG AGC AAG GGC GAG GAG-3′), and reverse primer (5′-ATA TTG GGA TCC TTA TTT CCA TTT CCA TTT TTT CGC CTT GTA CAG CTC GTC CAT GCC-3′) were used. Restriction sites included the NdeI and BamHI sites. Using standard subcloning procedures, FP-DBP sequence was inserted into the pET-15b vector and transformed into the *E. coli* BL21 (DE3) strains for protein expression. A single colony of the transformed cells was inoculated in a fresh LB media containing ampicillin. After inoculation of transformed cell, they were grown up to an optical density of ∼0.8 without IPTG. IPTG was used for induction and overexpression, and the transformed cells were inoculated in the media with a final concentration of 1 mM for IPTG. Following this, the cells for overexpression were kept on a shaker at a temperature less than RT for 24 hours, and cells for direct observations were collected at the desired time, which came an optical density over 2.0. For analysis of in vivo staining patterns, bacterial cells were mounted on slide glasses (quartz for DAPI, and glass for others). Cells for the protein purification were harvested by centrifugation at 12 000 × g, for 10 min (following centrifugations were performed under similar conditions), and the residual media was washed using the cell lysis buffer (50 mM Na_2_HPO_4_, 300 mM NaCl, 10 mM Imidazole, pH 8.0). Cells were lysed by ultrasonication for one hour and cell debris were separated by centrifugation. Ni-NTA agarose resin was added to the supernatants, and the mixture of the resin and cell proteins were kept on a shaker at 4°C for 6 h. The resin containing the bound protein was packed in an empty column for gravity chromatography, was further rinsed several times with protein wash buffer (50 mM Na_2_HPO_4_, 300 mM NaCl, 20 mM Imidazole, pH 8.0), and the bound proteins were finally eluted using the protein elution buffer (50 mM Na_2_HPO_4_, 300 mM NaCl, 250 mM imidazole, pH 8.0). After SDS-PAGE analysis, no further protein purification procedures were performed. Protein concentrations for the constructs were 10 mg/ml for eGFP, and 6 mg/ml for mCherry. All proteins were diluted (10 μg/ml for the results shown in Figure 1) using 50% (w/w) glycerol/1x TE buffer (Tris 10 mM, EDTA 1 mM, pH 8.0).

### DAPI nucleoid staining

*Escherichia coli* BL21 (DE3) cells were grown in fresh LB media at 37°C, until optical density 600 nm reached 1.0. Cells were harvested by centrifugation 12 000 × g, for 5 min. Supernatants were removed, and 0.85% NaCl solutions were used for re-suspension of cell pellets. Washing steps using 0.85% NaCl were repeated three times, finally followed by 1x TE or NaCl suspension. 4′,6-diamidino-2-phenylindole (DAPI) stock solution (5 mg/ml) was added at 20 μg/ml. After 30–60 min incubation at room temperature, cells were harvested again in order to remove unstained molecules. Suspended cells were mounted on quartz slide glass and coverslips.

### Glass surface preparation

The brief experimental procedure used for glass surface preparation used in Figure 1 is explained as follows (18). The glass coverslips were stacked in the Teflon rack, and soaked in piranha etching solution (30:70 v/v H_2_O_2_/H_2_SO_4_) for 2 h. After thorough rinsing of the coverslips with deionized water for several times, acid cleaned surface was used for protein coating. To adhere the positive charges on the surface, 200 μl of *N*-trimethoxymethylsilylpropyl-*N*,*N*,*N*-trimethylammonium chloride in 50% methanol was added to 200 ml of deionized water. The glass coverslips were incubated in this solution for 12 h, 65°C. These derivatized glass surfaces were used within 2 weeks of their preparation.

### Microchannel preparation

Fabrication of PDMS microfluidic devices followed standard rapid prototyping procedures as previously described (18). The patterns on a silicon wafer for microchannels (4 μm high and 100 μm wide) and nanoslit (450 nm high and 40 μm wide overlayed with 4 μm high and 80 μm wide microchannels were fabricated using soft lithography. The Cr mask was obtained from Amed Inc. (Seoul, Korea). SU-8 2005 photoresist (Microchem, Newton, MA, USA) was spin-coated onto the silicon wafer to make 4 μm high photoresist layer. After spin coating, the baked wafer was exposed under 350 nm irradiation. The patterned wafer was baked again and developed using a SU-8 developer (Microchem). The height was measured by a profilameter (Dektak XT, Bruker). The PDMS pre-polymer mixed with curing agent (10:1 weight ratio) was cast on the patterned wafer and cured at 65° C for 4 h or longer. Cured PDMS was peeled off from the patterned wafer and then PDMS devices were treated in an air plasma generator for 30 s with 100W (Femto Science Cute Basic, Korea) to make PDMS surface hydrophilic. PDMS devices were stored in water and air-dried before use.

### Fluorescence absorption/emission spectra

**Figure 2.**
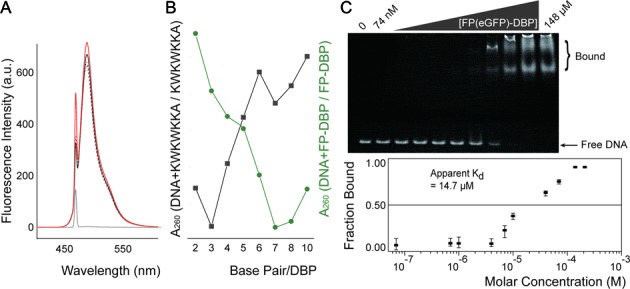
Characterization of FP-DBPs. (**A**) Fluorescence emission spectra of FP(eGFP)-DBP with oligonucleotide at 488 nm excitation. Spectra show fluorescence of FP(eGFP)-DBP with (red solid line) and without (black solid line) added oligodeoxynucleotides (0.5 μM, T_9_). As a control, eGFP without DBP (DNA-binding peptides) spectra were acquired in the presence and absence of oligodeoxynucleotides, shown here as dotted red and black lines, respectively. Gray solid line shows spectral data for just the oligonucleotide with no added FP-DBP. (**B**) Absorbance spectral ratios of DBP (KWKWKKA) with 52-mer DNA to DBP (grey) and FP-DBP with the same DNA to FP-DBP (green). The graphs indicate 3 base pairs per DBP, and 7 or 8 base pairs per FP-DBP. **(C)** Electrophoretic mobility shift assay (EMSA) estimates dissociation constant (*K_d_*) of FP(eGFP)-DBP. Analysis of polyacrylamide gel electrophoresis data reveals the mobility shift of double stranded 52-mer DNA / FP(eGFP)-DBP complex. EMSA experiments were repeated three times, and the error bars show the minimum and maximum values for each data set. Fraction bounds were analyzed using FIJI software, and *K_d_* was calculated by the least square method for each average value.

Emission spectra of the proteins and oligonucleotides were obtained with Hitachi F-7000 Fluorescence Spectrophotometer (Tokyo, Japan). Using emission spectrum mode at a fixed 488 nm excitation wavelength, intensity scans were performed from 300 to 900 nm. Final molar concentrations of eGFP and FP(eGFP)-DBP were 0.5 μM, and oligonucleotides were 0.5 μM as indicated. Dilution buffer and reference were 1x TE. Sequence of oligonucleotide used in this experiment was 5′-TTT TTT TTT-3′ (9 mer homopolymer oligonucleotide). Concentration adjusted samples were in fluorescence microcell 104.002F-QS (Hellma Analytics, Müllheim, Germany), and measured. For a measurement of absorbance ratio at a 260 nm wavelength, 52 mer double strands DNA (same as EMSA experiments) were used. Peptide and FP-DBP were diluted corresponding to bp:dye ratios used in Figure 2. Concentrations were 5 μM for DNA stock solutions, and DNA solutions were mixed as 1:1 (v/v) with diluted peptide/FP-DBPs. Concentration adjusted samples were in fluorescence μCuvette (Eppendorf, Hamburg, Germany), and measured using BioPhotometer Plus (Eppendorf).

### Electrophoretic mobility shift assay

A 52-mer random sequence oligonucleotide (5′-CTA CTA GCA CAA TCG ACT GTA CGG ACC GAT CGA GTC ACT AGC AGT CTA GCA A-3′) and its complementary sequence oligonucleotide are hybridized into double strands DNA. The same molar concentrations of two oligonucleotides in a microcentrifuge tube were soaked in boiled water, followed by cooling down to the room temperature. Hybridized double strands DNA oligo was diluted to 0.5 ng/μl (∼31.4 nM) using 1xTE buffer. FP(eGFP)-DBP was diluted with 50% (v/v) glycerol/ 1x TE, and corresponding molar concentrations are 74 nM, 740 nM, 1.48 μM, 3.7 μM, 7.4 μM, 14.8 μM, 37 μM, 74 μM and 148 μM, respectively. The diluted proteins, DNA oligomer, and 50% (v/v) glycerol/1x TE buffer to adjust total reaction volume (10 μl) were added to each sample tube. Each sample was loaded into 0.5x TBE 4–20% polyacrylamide precast gel (TBE–PAG). After running 75 min at 151 V constant, the gel was stained with EtBr for 5 min, and was destained excess EtBr in deionized water for several minutes. Images of retarded DNA were captured with CCD camera (WGD-20, Daihan Scientific Co., Korea) on UV transilluminator (WUV-M20, Daihan Scientific Co., Korea). Fraction bounds of each lane were represented as intensity profiles of bound proteins/free DNA, and analyzed with ImageJ Gel Analyze. Apparent dissociation constants of protein concentrations were calculated as *K*_d_ = ((1-*f*)/*f*)[protein]_total_ where *f* is the bound fraction as shown in Figure 2.

### Photocleavage of DNA

**Figure 3.**
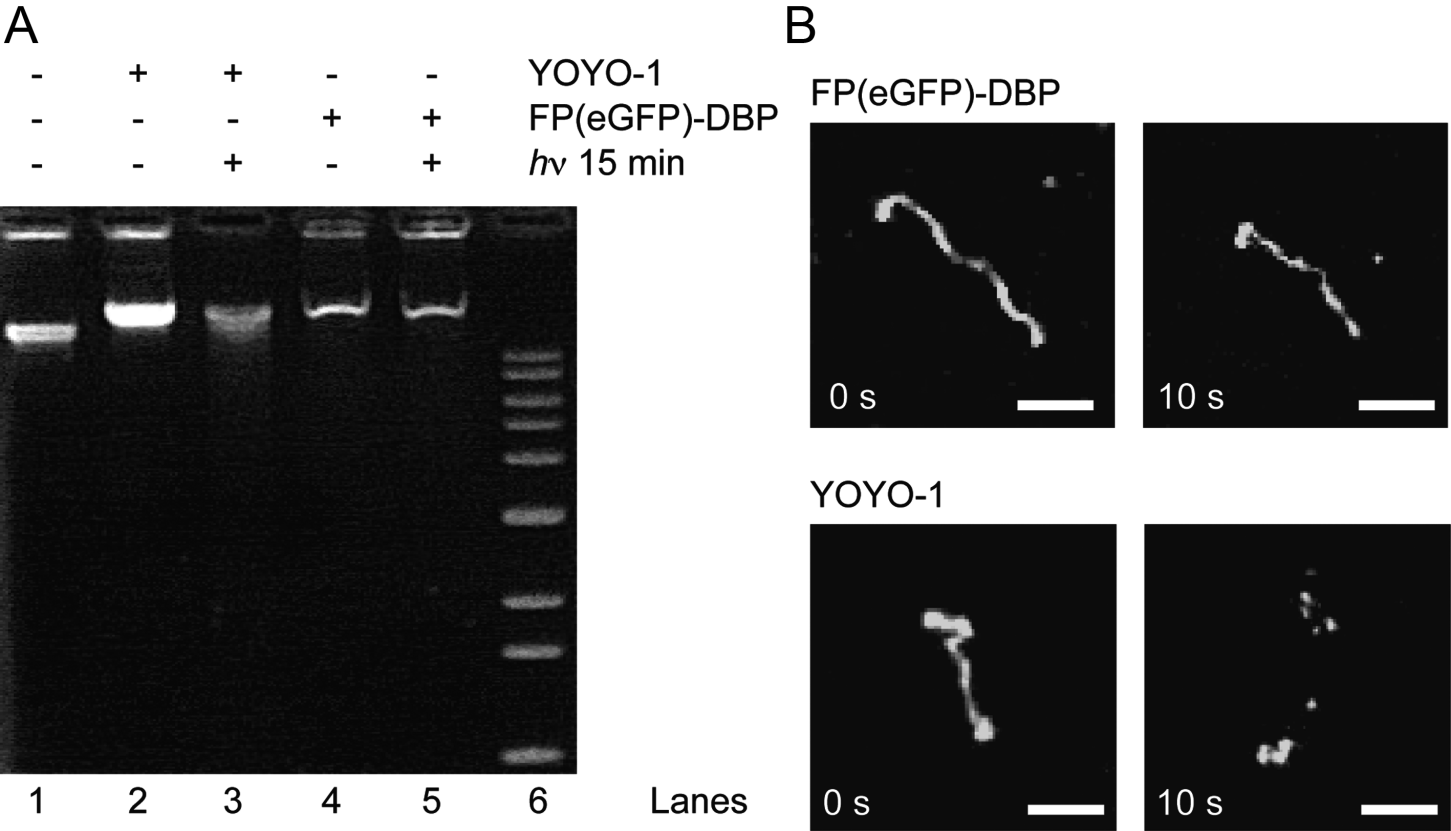
Laser induced photocleavage of large DNA molecules. (**A**) Photocleavage products separated by 1% agarose gel electrophoresis. Lane 1: Control λ DNA (48.5 kb). Lane 2: YOYO-1 stained λ DNA. Lane 3: YOYO-1 stained λ DNA exposed to a 488 nm laser source for 15 min. Lane 4: FP(eGFP)-DBP stained λ DNA. Lane 5: FP(eGFP)-DBP stained λ DNA exposed to 0.71 W/cm^2^ of a 488 nm laser source for 15 min. Lane 6: 1 kb ladder. (**B**) Fluorescence micrographs T4 DNA molecules (166 kbp within slits (40 μm x 80 μm x 450 nm) stained with FP(eGFP)-DBP, and YOYO-1. After 10 s of 488 nm laser illumination, YOYO-1 stained T4 DNA molecules were cleaved, whereas FP-DBP (eGFP) stained molecules remained intact. Both samples were exposed to 5.38 mW/cm^2^. Scale bars = 5 μm (*see* Supplementary Movie 1).

For an agarose gel analysis in Figure 3a, λ DNA (0.5 mg/ml) were diluted 10 times with 1x TE buffer. Each sample was mixed as 1:1 (v/v) with appropriate staining molecules: 1× TE for control λ DNA, 19.2 μM of YOYO-1, and 25.7 μM of FP(eGFP)-DBP, which were 4 bp/dye ratio for YOYO-1, and 3 bp/dye for FP-DBPs. Mixed samples in thin-wall Eppendorf tubes were incubated 30 min at RT. Induced 488 nm laser source was identical with fluorescent microscope. Exposed intensity was 0.71 W/cm^2^. Each sample was mixed with 6× gel loading dye, and loaded in freshly made 1% of agarose in 0.5× TBE. After running 30 min at 130 V constant, the gel was stained and illuminated in the same manners. For single molecule experiments in Figure 3b, the edges of coverslips on a square acryl holder were sealed with paraffin wax, and the PDMS nanoslit device was mounted on the coverslips. After mounting, T4 DNA (0.25 mg/ml) with 1 μM YOYO-1 and 300 nM of FP-DBP was loaded into the nanoslit *via* applied electrical field (30 V across 25 mm) (19). For single molecule DNA experiment, exposed intensity was 5.38 mW/cm^2^, measured at the image plane using a power and energy meter (PM100D, Thorlabs, Newton, NJ, USA).

### Flow chamber

A flow chamber was prepared by placing a cover slip on the glass slide with a spacing of 100 μm between the two, using a double-sided tape as previously described (20,21). The inlet and outlet holes were drilled on this microscope glass slide using a diamond-coated bit. A yellow pipette tip was installed in the inlet port and a tubing line was connected to the outlet port with an epoxy bonding that was cured at room temperature for 5 min. Further, the cover slip was fixed on the glass slide using a double-sided 3M tape. The dimensions of the flow chamber were 3 × 17 × 0.1 mm (*L* × *W* × *H*) and the total volume of the flow chamber was 5.1 μl. A syringe pump, NE-1000 (New Era Pump Systems Inc., Wantagh, NY, USA), was used to control the buffer delivery into the flow cell with a flow rate of 0.150 ml/min that corresponded to 8.3 mm/s.

### Protein surface preparation

**Figure 4.**
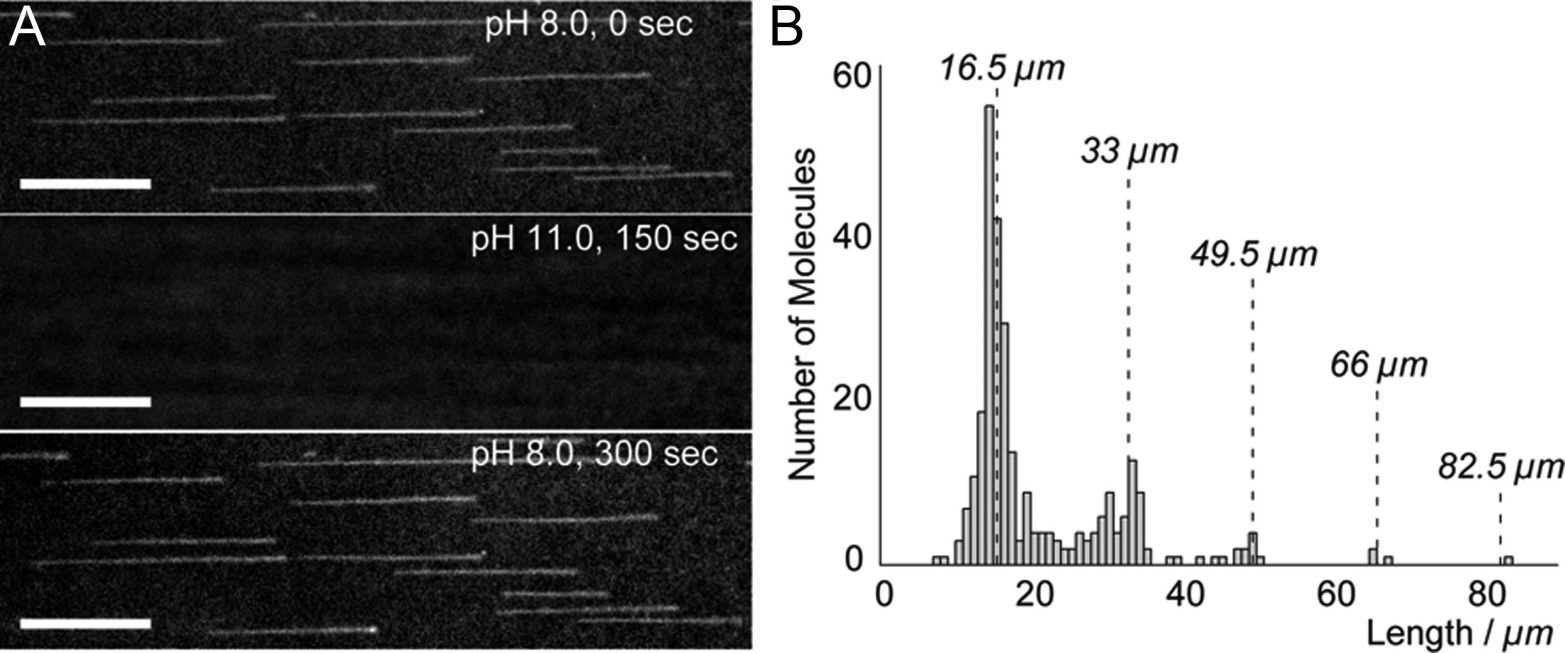
FP-DBP staining is pH reversible and does not perturb DNA polymer contour length. (**A**) Fluorescence microscopy images of biotin-labeled λ DNA showing reversible staining of eGFP-DBPs by changing the pH of buffers within a flow chamber. Scale bars represent 10 μm (*see* Supplementary Movie 2). (**B**) Histogram of the contour lengths of stretched λ DNA and its concatemers, which matches the theoretical contour lengths of λ concatemers (48 502 bp × 0.34 nm/bp = 16.5 μm), as represented by the dotted lines.

The surface of the flow cell used in Figure 4 was initially coated using biotin-BSA followed by neutravidin, as previously described (20). The biotin-BSA stock solution (10 mg/ml, 10 mM Tris, 50 mM NaCl, pH 8.0) was diluted 10 times with 10 mM Tris buffer and then loaded in the flow chamber for coating. After 5 min of BSA adsorption, neutravidin solution (0.25 mg/ml 10 mM Tris, 50 mM NaCl, pH 8.0) was loaded into the BSA-coated flow chamber. The set-up was kept at room temperature for 5 min. After 5 min of neutravidin adsorption, the flow chamber was further coated with a hundred times diluted BSA stock solution (1× TE buffer). The flow chamber setup was again kept at room temperature for 5 min.

### Reversible staining

After the preparation of neutravidin-coated surfaces in Figure 4, 1 μM of λ DNA overhang oligo (5′-pGGGCGGCGACCT-TEG-biotin-3′) were loaded into the flow chamber, and kept at RT for 5 min. The λ DNA, T4 DNA ligase, and the reaction buffer were added, and kept at RT for 30 min. After washing the residual enzyme mixture, 1 μM of complementary λ DNA overhang oligo conjugated with fluorescent quencher BHQ-1 (5′-pAGGCCGCCGCCC-BHQ1-3′) were loaded into the flow chamber, and kept at RT for 30 min. The diluted FP(eGFP)-DBP flowed into the channels resulting in the visualization of the tethered DNA. For the purpose of destaining of the attached FP-DBP, 50 μl of TE buffer (adjusted to pH 11.0) was loaded in the flow chamber. The residual buffer was immediately washed 200 μl of TE buffer (pH 8.0) and then diluted proteins were loaded. Visualization of stained DNA molecules was done under a continuous flow of the diluted FP-DBP with a flow rate maintained at 100 μl/min.

## RESULTS AND DISCUSSION

### DNA staining of FP-DBPs and characterization

Figure 1 shows the green (eGFP) and red (mCherry) stained DNA molecules using these FP-DBPs (fluorescent protein–DNA binding peptides). The FP-DBPs confluently bind DNA molecules *via* two small DNA-binding peptides (KWKWKKA) attached to both the C- and N-termini of these fluorescent proteins. The design for this DNA-binding peptide sequence was motivated by our previous report that suggested that neutravidin-coated surfaces bind DNA molecules in a reversible manner (20). In this publication, we reported that the protruding tryptophan and lysine (WK) residues formed a key component for immobilizing DNA molecules on the neutravidin surface. In a series of studies (22,23), dated back to the 1970s, Helene *et al*. pioneered the understanding of how tryptophan- and lysine-containing-peptides bind single or double stranded DNA. They reported a two-step DNA binding mechanism for such peptides: (i) the positively charged lysine residues show electrostatic interactions with the negatively charged phosphate backbone of the DNA molecules; and (ii) the aromatic ring of the tryptophan residue intercalates within the adjacent bases on the DNA. They also reported that the binding affinity of the KWK motif on DNA is altered by the environmental pH (i.e. becoming very weak when the pH > 8.5) (24). We leveraged this important effect for conferring pH control of binding and dissociation of our FP-peptide constructs (FP-DBPs), which allow simple conditions for DNA staining and destaining. We then optimized the DNA binding peptide sequence by testing sequences such as KWKKA, (KW)_2_KKA, and (KW)_5_KKA linked to the enhanced GFP (eGFP) at N-terminus, or to both the C- and N-termini (*see* SI for more details). In order to make the binding affinity comparable to organic dyes such as EtBr or YOYO-1, we gradually increased the number of KW repeats in the peptides studied. Mascotti and Lohman characterized several variations of these peptides such as K_5_, KWK_4_, KWK_3_WK and (KW)_3_KK, each of which contained five lysine residues with increasing number of tryptophan residues (25). They observed that the presence of more number of tryptophan residues lead to higher association constant, which can be calculated using the number of tryptophan residues present. The KWKWKKA peptide present on both ends of the eGFP provided clear DNA images stained with green colored fluorescence using epifluorescence microscopy, as shown in Figure 1. The concentration of FP-DBP we used was 300 nM for Figure 1, but DNA molecules could still be imaged at labeling concentration down to 50 nM. Alternatively, we also constructed a red FP-DBP using the mCherry protein with the same binding peptide, as shown in Figure 1b.

In addition, fluorometer measurements showed slightly increased luminosity for FP(eGFP)-DBP/DNA complexes, as compared to free eGFP with the same amount of DNA present, as shown in Figure 2a. Despite of the slight increase, the primary fluorescence emission comes not from intercalations but from fluorescent proteins. Generally, intercalation dyes such as YOYO-1 and EtBr has the property that fluorescent emission is significantly enhanced when they are intercalated into DNA. Unfortunately, FP-DBP does not have this typical advantage of intercalation dyes since tryptophan intercalation is not related with fluorescent protein emission. According to the intrinsic brightness of fluorescent proteins, excessive concentrations of FP-DBP may enhance the background noises; therefore, staining conditions should be carefully adjusted. In order to determine the number of base pairs (bp) per single FP-DBP, we measured absorbance spectra of oligonucleotide (52-mer double strands) with FP-DBPs at 260 nm, since it is kwown that DNA-peptide (KWKWKKA) complexes quench the absorbance of DNA at 260 nm (26). Figure 2b implies that the number of base pairs per FP-DBP is 7 or 8 bp/FP-DBP, whereas that of KWKWKKA is 3 bp/DBP. The dissociation constant, *K*_d_, for the FP(eGFP)-DBP with double strand DNA was determined to be 14.7 μM, using the electrophoretic mobility shift assay, as shown in Figure 2c. The *K*_d_ of FP-DBP is comparable to EtBr (*K*_d_ = 12.1 μM), but is significantly weaker than that of YOYO-1 (*K*_d_ = 12.1 nM) (27,28). These new protein reagents, which confluently stain DNA molecules, offer numerous advantages over their dye counterparts such as EtBr, YOYO-1 and SYTO: (i) FP-DBPs are safe for those who deal with DNA staining dyes. Owing to their steric mass, it is impossible for FP-DBPs to diffuse into the human cells unlike chemical dyes—most organic dyes are considered as potential mutagenic compounds. (ii) Simple plasmid constructs produce the FP-DBPs with a range of spectral qualities, and present opportunities for controllable expression within transformed cells. (iii) FP-DBPs are readily incorporated within experiments involving live cells or living organisms. In addition to the above-mentioned advantages of fluorescent proteins, FP-DBPs offer additional benefits compared to fluorescent organic dyes.

### Photo-cleavage events

The most notable advantage of FP-DBPs for staining DNA is that they do not mediate DNA photocleavage, which makes them ideal staining reagents for imaging of DNA molecules over extended time periods. As shown in Figure 3 (*see* Supplementary Movie 1a), DNA staining with organic dyes commonly leads to DNA breakage because illuminated fluorophores continually cycle through excitation and emission, which greatly enhances photochemical reactions causing DNA photocleavage (17,29). Accordingly, DNA fluorescence studies involving organic dyes require careful illumination dosages and copious addition of anti-bleaching agents (e.g. 4% v/v β-mercaptoethanol in a buffer solution), particularly within microfluidic shear-induced flows (30). Furthermore, dye-induced photocleavage of DNA is also problematic for *in vivo* experiments, because the photocleavage leads to critical damages to the live cells. In contrast, FP-DBPs do not induce photocleavage problem even without anti-bleaching agent because the DNA binding moiety (e.g. KWKWKKA) of the FP is separated from the fluorophore moiety, which is generally buried within a β-barrel (*see* Supplementary Movie 1b). Thus, there is very little opportunity for the fluorophore to react with the DNA molecules. More specifically, the absence of dye-mediated photocleavage inherent to FP-DBPs is critically advantageous for use in DNA experiments involving very large, tethered, or freely diffusing molecules because such molecules are easily broken by few photocleavage events.

### Reversible staining of FP-DBPs *via* pH shift

Another important advantage of FP-DBPs is controllable staining and de-staining *via* pH shifts. Figure 4a demonstrates the reversible DNA staining using FP (*see* Supplementary Movie 2). In this experiment, DNA molecules were stained with FP(eGFP)-DBP at pH 8.0, and then washed free of FP-DBP at pH 11.0. After this wash, the FP-DBPs were reloaded into the device to re-stain DNA molecules at pH 8.0. This reversible binding capability allows bleached FP-DBPs to be supplanted by fresh ones, thus potentiating experiments requiring extended imaging times. Notably, it was possible to image DNA without noticeable dissociation of FP-DBP for 30 min, which provides sufficient time to visualize single DNA molecules. In addition, we could control DNA staining and destaining by FP-DBPs by other reagents. For example, we observed that FP-DBPs were dissociated from DNA in high salt buffer condition (50 mM potassium acetate, 10 mM magnesium acetate, 20 mM Tris–acetate). We also observed that addition of anionic detergent (1% v/v *N*-nauroylsarcosine) could remove FP-DBPs from DNA, which could be re-stained after 1× TE washing and fresh FP-DBP loading. Furthermore, we observed that tethered DNA stained with eGFP-DBPs was gradually replaced by red colors with continuous buffer flows containing mCherry-DBPs or vice versa (11).

It is also noteworthy that the FP-DBPs do not alter the contour length of double stranded DNA, while most of the intercalating dyes, such as EtBr and YOYO-1, are known to distort the DNA structure and increase its full contour length (31,32). For example, we measured the full contour length of tethered λ DNA molecules, from Figure 4a, that were fully stretched by a microfluidic flow. Figure 4b shows that the λ DNA monomer is 16.5 μm long, which matches the theoretically expected value of 48 502 bp × 0.34 nm/bp = 16.5 μm. This result markedly differs from our previous contour length measurements for λ DNA molecules (22 μm) that were stained with YOYO-1 and tethered on surfaces (20). This result implies that the FP-DBP does not appreciably alter DNA molecular structure in spite of intercalations, which is typically known to distort DNA structure due to deep insertions into stacked planar structures. Our observation is further supported by a previous study by Desoye and Porschke showing that DNA contour length did not increase even after tryptophan intercalation because its indole ring was only partially inserted within stacked DNA bases (33).

### Bacterial nucleoid *in vivo* staining

**Figure 5.**
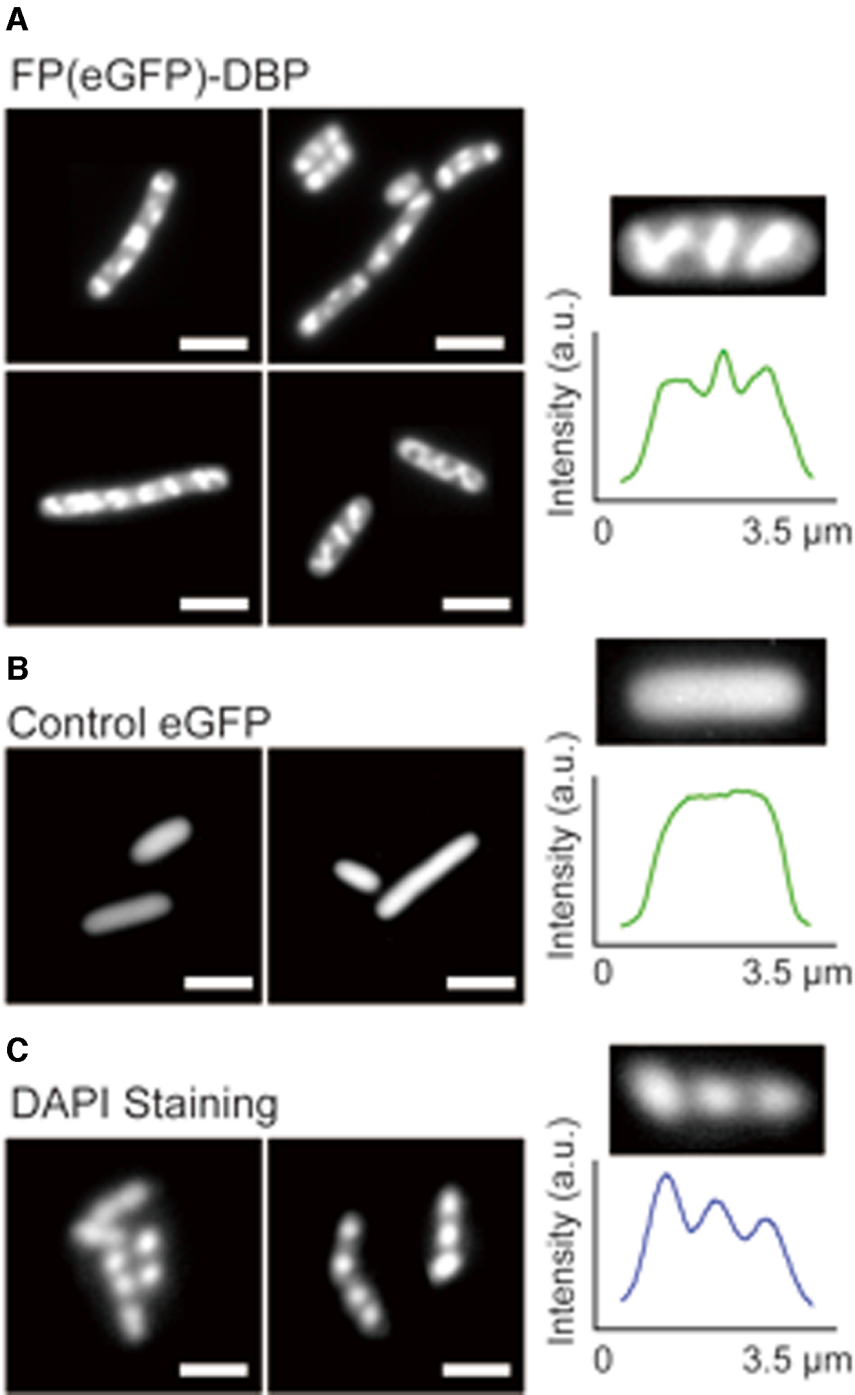
Live *E. coli* cells are specifically stained by endogenously expressed FP-DBP. (**A**) *E. coli* cells (BL21) expressing FP(eGFP)-DBP show discrete patterns within bacterial cells. Images show localized fluorescence features within *E. coli* cells; a fluorescence intensity profile across one cell details this pattern. (**B**) Control: *E. coli* cells expressing eGFP, but *without* the KWKWKKA peptide, and its *featureless* fluorescence intensity profile. (**C**) *E. coli* cells stained with DAPI, showing similar patterns of nucleoids. Scale bars = 5 μm.

As previously discussed, the most significant advantage of FP-DBPs for DNA staining is its application to live cells, or entire living organisms. Figure 5 illustrates the DNA-staining FPs expressed within bacterial cells (*E. coli* BL21). In comparison with unmodified eGFP, FP(eGFP)-DBP presents distinct patterns within the growing cells. In a unique fashion, the FP(eGFP)-DBP fluorescence localized in three or four discrete regions within the bacterial cells: one, or two spots in the middle, and some at both ends of cells, as well as DAPI stained bacterial cells. These fluorescent punctates may correspond to observations made by Lemon and Grossman, who showed, using a GFP-tagged DNA polymerase that replication forks anchored in the middle of the cell separated during growth (34). Moreover, Berlatzky *et al*. observed that the newly synthesized DNA moves *via* a helical path from the middle of the cell towards two poles, where the DNA accumulates (35). Furthermore, we observed cell growth rates after expressing FP-DBP (*see* SI Supplementary Figure S1). Although it was relatively slow, FP-DBPs did not totally inhibit the cell growth, implying that bacterial cells can still grow and divide even after considerable amount of FP-DBPs are expressed in a cell.

## CONCLUSION

Imaging confluently stained DNA molecules is complicated by issues of photodamage and phototoxicity mediated by organic dyes. Although fluorescent proteins do not generally suffer such issues and are well-targeted by many fusion techniques to cellular and molecular structures, they do not offer the ubiquitous binding patterns inherent to simple dyes. As such, we combined the virtues of organic and protein fluors by creating fluorescent fusion proteins (FP-DBP) featuring tightly binding peptides that promiscuously cover DNA molecules and when expressed within *E. coli* cells enabled the localization of nucleoid structures. Controllable expression of FP-DBPs will likely foster other new routes to localization of nucleic acids within living cells and offers yet another important advantage over organic dyes. Aside from reversible staining *via* pH shifts, we have also demonstrated FP-DBP confluent staining of naked DNA molecules in ways comparable to organic dyes, but featuring minimal photodamage during irradiation, and minimal perturbation of the DNA polymer contour length.

## Supplementary Material

SUPPLEMENTARY DATA
