# Supplementary material for: DNA binding fluorescent proteins for the direct visualization of large DNA molecules
Source: Nucleic Acids Res. 2015 Aug 11;44(1):e6. doi: 10.1093/nar/gkv834 (PMC4705684; doi:10.1093/nar/gkv834)
Supplement: SUPPLEMENTARY DATA [file supp_44_1_e6__index.html]

DNA binding fluorescent proteins for the direct visualization of large DNA molecules — DNA binding fluorescent proteins for the direct visualization of large DNA molecules — SUPPLEMENTARY DATA 

# DNA binding fluorescent proteins for the direct visualization of large DNA molecules

## SUPPLEMENTARY DATA

- SUPPLEMENTARY DATA
- SUPPLEMENTARY DATA
- SUPPLEMENTARY DATA
