# Supplementary material for: DNA binding fluorescent proteins for the direct visualization of large DNA molecules
Source: Nucleic Acids Res. 2015 Aug 11;44(1):e6. doi: 10.1093/nar/gkv834 (PMC4705684; doi:10.1093/nar/gkv834)
Supplement: SUPPLEMENTARY DATA [file supp_gkv834_nar-01609-met-k-2015-File007.docx]

**DNA Binding Fluorescent Proteins for the Direct Visualization of Large DNA Molecules**

Seonghyun Lee^1^, Yeeun Oh^1^, Jungyoon Lee^1^, Sojeong Choe^1^, Sangyong Lim^2^, Hyun Soo Lee^1^, Kyubong Jo^1*^, and David C. Schwartz ^3^

**Supplementary Information (SI)**

**Supplementary Data**

**Optimization of DNA-binding Peptide**

Since a single moiety of KWK have an ability to bind single and double strands DNA ([1](#_ENREF_1),[2](#_ENREF_2)), we could roughly characterize following candidates in Table S1, which were constructed simply by repeating lysine and tryptophan residues, KWK-KA. KA was used for short, and non-helical linker amino acids. Despite of binding evidences in NMR, single DNA molecules were not visualized with KWK-KA-eGFP, assuming that it has somewhat different binding aspects between oligonucleotides-peptides and large DNA-protein constructs. Therefore, we had decided to repeat the KW- moiety at the N-terminus, i.e. KWKWKWKWKWKKA-eGFP. This peptide moiety has sufficient ability to bind onto DNA, yet bright spots appeared, which was shown as protein aggregations, even in 50% glycerol/1x TE. To avoid nonspecific protein-protein interactions, we symmetrically tagged the moiety -AKK(WK)_5_ at C-terminus to flap. However, 5 times repeated KW moiety could not stain any DNA single molecule, assuming that increased positively charged amino acid (lysine) might interrupt the assembly of proteins on DNA, due to strong electrostatic repulsions of each proteins. Testing the candidates from Table S1, we clarified that (KW)_2_KKA at both N- and C-termini is the most efficient binding moiety.

**Table S1. List of Peptides Used in Optimization**

|  | N-Terminal | C-Terminal | Result |
| --- | --- | --- | --- |
| 1 | KWKKA | - | Insufficient Binding Affinity; Lack of Binding Property with KWK Single Moiety |
| 2 | (KW)_5_KKA | - | Visualized DNA with Undesirable Noises from Protein Aggregations |
| 3 | (KW)_5_KKA | AKK(WK)_5_ | Strong Repulsions between FP-DBPs; Not Visualized |
| 4 | (KW)_2_KKA | AKK(WK)_2_ | Observation of Properly Stained DNA |

**
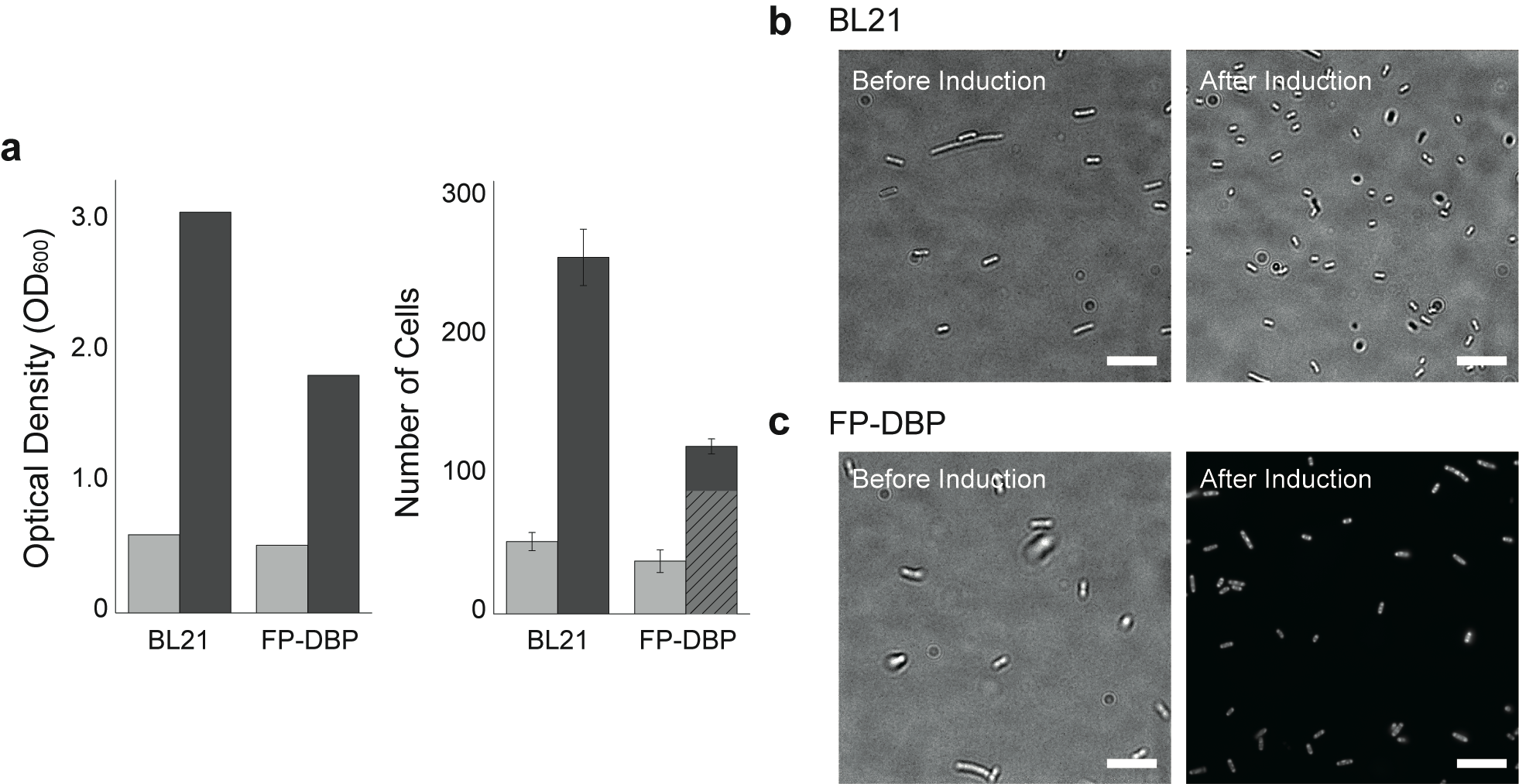
**

**Figure S1. Cell division rates of FP-DBP expressing *E. coli*. a)** Cell growth of control *E. coli* BL21 cells and FP-DBP expressing *E. coli*. Cells before IPTG induction are shown in light grey, and cells after 12 hours from the induction are in dark grey. The left graph shows optical density values at 600 nm to indicate overall growth rates of cells. The right graph represents the number of bacterial cells that we counted from microscopic images (b, c), and error bars show the standard deviation from five images. In FP-DBP data, bar with diagonal lines represents glowing *E. coli* with fluorescent emission, and the other shows unglowing *E. coli* without fluorescent emission. **b)** Representative microscopic images for control *E. coli* BL 21 without FP-DBP **c)**. Representative microscopic images for FP-DBP expressing *E. coli* BL 21 before and after induction (12 hours after adding IPTG). Scale bars are 10 µm.

**Movies**

**Supplementary Movie 1. Comparison of DNA photocleavage events, in free solution, stained with YOYO-1, or FP(eGFP)-DBP.** T4GT7 DNA molecules (166 kb) are confined and stretched within a thin slit (40 µm x 80 µm x 450 nm) and illuminated by 488 nm-laser source of 50 mW (2.5 mW at the objective lens). DNA molecules are stained with: (a) YOYO-1, with added β-mercaptoethanol (4 % v/v) to attenuate photocleavage and bleaching in 1x TE (10 mM Tris and 1 mM EDTA pH 8.0) (b) DNA bound with FP(eGFP)-DBP even without added β-mercaptoethanol in 1xTE.

**Supplementary Movie 2. Reversible staining *via* pH shifts of FP(eGFP)-DBP bound to a tethered λ DNA concatemer.** DNA molecules were first bound with FP(eGFP)-DBP and then destained by shifting the pH from 8.0 to 11.0. The pH was then shifted back to 8.0, for allowing restaining, after washing with pH 8.0 1xTE buffer. Movie annotations show reaction conditions and frame playback rates.

1. Montenay, T. and Helene, C. (1968) Molecular Interactions between Tryptophan and Nucleic Acid Components in Fronzen Aqueous Solutions. *Nature*, **217**, 844-845.

2. Helene, C. and Dimicoli, J.L. (1972) Interaction of oligopeptides containing aromatic amino acids with nucleic acids. Fluorescence and proton magnetic resonance studies. *FEBS Lett*, **26**, 6-10.
